# The Pink Rim Sign: Location of Pink as an Indicator of Melanoma in Dermoscopic Images

**DOI:** 10.1155/2014/719740

**Published:** 2014-02-03

**Authors:** Ryan K. Rader, Katie S. Payne, Uday Guntupalli, Harold S. Rabinovitz, Maggie C. Oliviero, Rhett J. Drugge, Joseph J. Malters, William V. Stoecker

**Affiliations:** ^1^Stoecker & Associates, LLC, 10101 Stoltz Drive, Rolla, MO 65401-7714, USA; ^2^Missouri University of Science & Technology, G20 Emerson Electric Co. Hall, Rolla, MO 65409-0040, USA; ^3^Skin and Cancer Associates, 201 NW 82nd Avenue, Bennett Medical Plaza, Suite 501, Plantation, FL 33324, USA; ^4^Sheard & Drugge PC, 50 Glenbrook Road, Unit 1C, Stamford, CT 06902, USA; ^5^The Dermatology Center, LLC, 10101 Stoltz Drive, Rolla, MO 65401, USA

## Abstract

*Background*. In dermoscopic images, multiple shades of pink have been described in melanoma without specifying location of these areas within the lesion. *Objective*. The purpose of this study was to determine the statistics for the presence of centrally and peripherally located pink melanoma and benign melanocytic lesions. *Methods*. Three observers, untrained in dermoscopy, each retrospectively analyzed 1290 dermoscopic images (296 melanomas (170 *in situ *and 126 invasive), 994 benign melanocytic nevi) and assessed the presence of any shade of pink in the center and periphery of the lesion. *Results*. Pink was located in the peripheral region in 14.5% of melanomas and 6.3% of benign melanocytic lesions, yielding an odds ratio of 2.51 (95% CI: 1.7–3.8, *P* < 0.0001). Central pink was located in 12.8% of melanomas and 21.8% of benign lesions, yielding an odds ratio of 0.462 (95% CI: 0.67, *P* = 0.204). Pink in melanoma *in situ* tended to be present throughout the lesion (68% of pink lesions). Pink in invasive melanoma was present in 17% of cases, often presenting as a pink rim. *Conclusions*. The presence of pink in the periphery or rim of a dermoscopic melanocytic lesion image provides an indication of malignancy. We offer the “pink rim sign” as a clue to the dermoscopic diagnosis of invasive melanoma.

## 1. Introduction

Any pink color within a melanocytic lesion can be regarded as a marker for inflammation and has long been recognized as a clinical clue to melanoma [1, 2]. In melanomas without significant pigmentation, either amelanotic or hypomelanotic, a subset of these dermoscopically visible areas has been called “milky-red areas” [3–5]. Johr published a series of cases that characterized amelanotic melanoma with pink regions [2]. Menzies et al. listed more than one shade of pink being a statistically significant positive indicator of melanoma (odds ratio = 2.3, sensitivity = 32.4%, and specificity = 82.9%) versus all nonmelanomas [5].

Our goal in this study was to see if the presence of pink can be detected, even by those unskilled in dermoscopy, with the goal of earlier identification of melanoma. In this research, the term “pink” is not defined other than the color pink as understood by dermoscopically naïve observers. We sought additionally to determine whether location of pink in the lesion is important. Preliminary analysis determined that pink located in the periphery of dermoscopic lesions could assist in early detection of melanoma.

## 2. Methods

The image set used in this study consists of 1290 digital, contact nonpolarized dermoscopic images of melanocytic lesions, acquired during the National Institutes of Health-funded study SBIR R44 CA-101639-02A2. These contact images were obtained with gel interface and minimal pressure. This image set included 296 invasive malignant melanomas and melanomas *in situ* and 994 benign melanocytic lesions including 461 dysplastic nevi with mild atypia, 70 dysplastic nevi with moderate atypia, 271 nevocellular nevi without atypia, and 192 congenital nevi. Images were acquired at four clinics: Skin and Cancer Associates (Plantation, FL), the Dermatology Center (Rolla, MO), Columbia Dermatology (Columbia, MO), and Sheard and Drugge PC (Stamford, CT). The images had a resolution of 1024 × 768 pixels. The Phelps County Regional Medical Center Institutional Review Board (Rolla, Missouri) approved this research and each subject or subject's parent or guardian signed a consent form for this research. All melanomas were biopsied and examined by a dermatopathologist and all benign lesions were either biopsied or followed and determined to have no change. The high *in situ* to invasive melanoma ratio (1.35) reflects the detection of melanomas at an early stage in four dermatology private practices in the USA [6]. Lesions were included if they were biopsied or if either patient or physician suspected the possibility of melanoma. The NIH study lesions comprised a convenience sample of all lesions seen in these four clinics. All melanocytic lesions in this NIH study were included in the current study except the following classes: Spitz-Reed nevi, nevi with known previous treatment (nevus recurrens), lesions with multiple diagnoses/collision tumors, and lesions with high-grade atypia, blue nevi, and metastatic melanomas.

Each image was analyzed without knowledge of diagnosis by three untrained observers: two premedical students with eight months' clinical research experience finding borders for lesions in dermoscopic images (RKR, KSP) and an engineering graduate student (UG) with no clinical experience and six months of image processing experience. The three observers independently determined if any peripherally located or centrally located pink areas were present. Consensus agreement was obtained after individual assessments were completed. The study was supervised by a dermatologist with 18 years of experience in dermoscopy (WVS).

**Figure 1:**
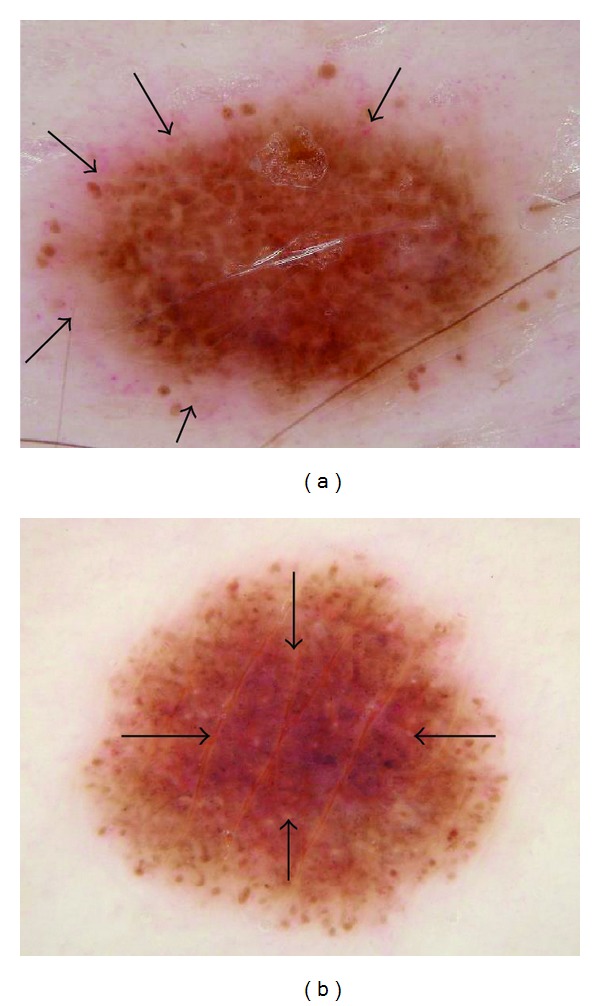
(a) *In situ* melanoma arising within a congenital lesion, 4 mm, abdomen, 34-year-old male. Peripheral pink with some eccentric pink centrally: *in situ* melanoma, 4 mm wide, right abdomen, arising within a superficial compound congenital nevus; pink rim sign is present (arrows) just external to irregular network lacking pink. Note negative network and peripheral globules that are fairly uniform and symmetric. (b) Pink throughout lesion: benign nevus, not biopsied, 5 mm nevus on upper arm in 35-year-old female; pink is present at rim but is most prominent centrally (arrows). Pink rim sign is absent. Note similar architecture and symmetrically arranged globules in both lesions.

**Figure 2:**
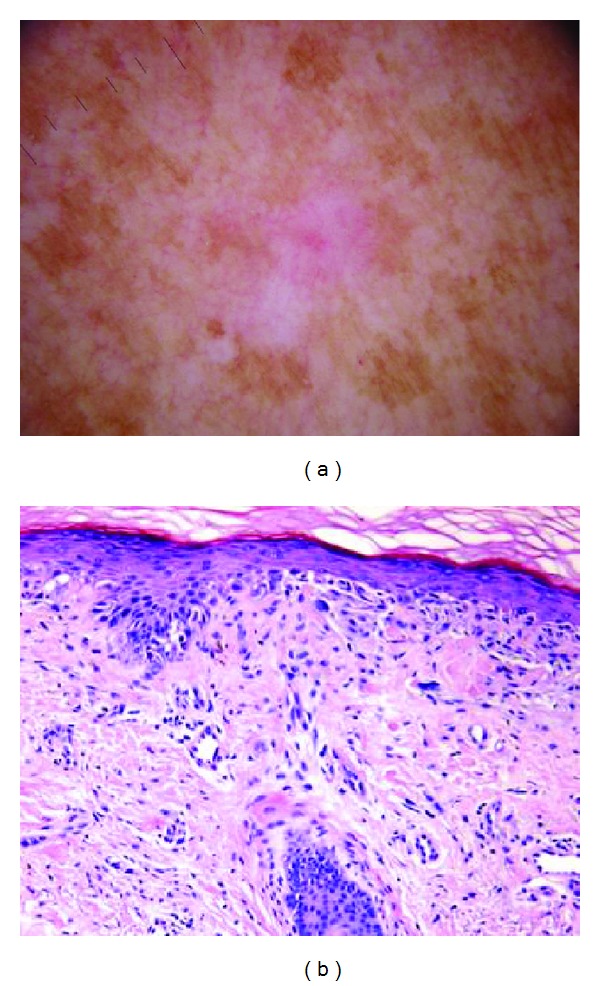
(a) Noncircumferential peripheral and central pink: completely amelanotic invasive melanoma, on right upper arm, 42-year-old female, depth 0.55 mm, 5 mm wide; patient had not noticed the lesion. Pink is present; pink rim sign is absent. (b) Histopathology for melanoma.

Pink coloration can be present in various shades. We offer several examples of pink, including light pink (Figures 1 and 2), dark pink (Figure 4(a)), and pink-orange (Figure 5(a)). We did not demand any particular shade or any medical diagnosis, such as erythema, but rather sought to include all lesions for which the untrained observers agreed that there was some shade of pink present. For people with fair skin, with an identifiably pink background, pink must be increased from the background, as in Figures 1(a) and 1(b). The lesion was not considered pink if there was no change in pink from the background skin color.

**Figure 3:**
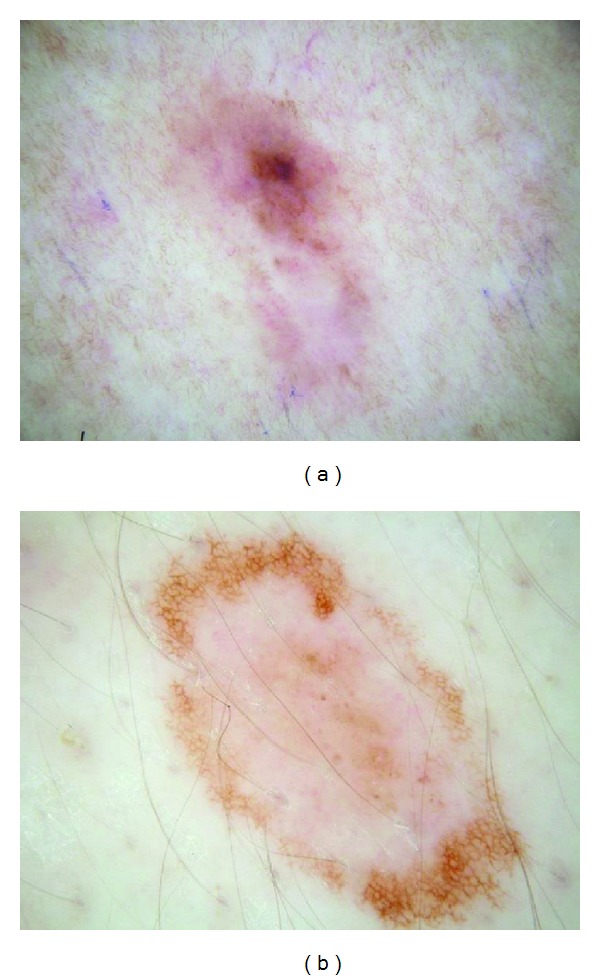
(a) Invasive melanoma, 8 mm wide, back, 62-year-old female, depth 0.55 mm. Nearly circumferential peripheral and central pink. (b) Clark compound nevus, 9 mm wide, 17-year-old male, biopsy confirmed. Central-only pink. Pink rim sign is absent in both (a) and (b).

**Figure 4:**
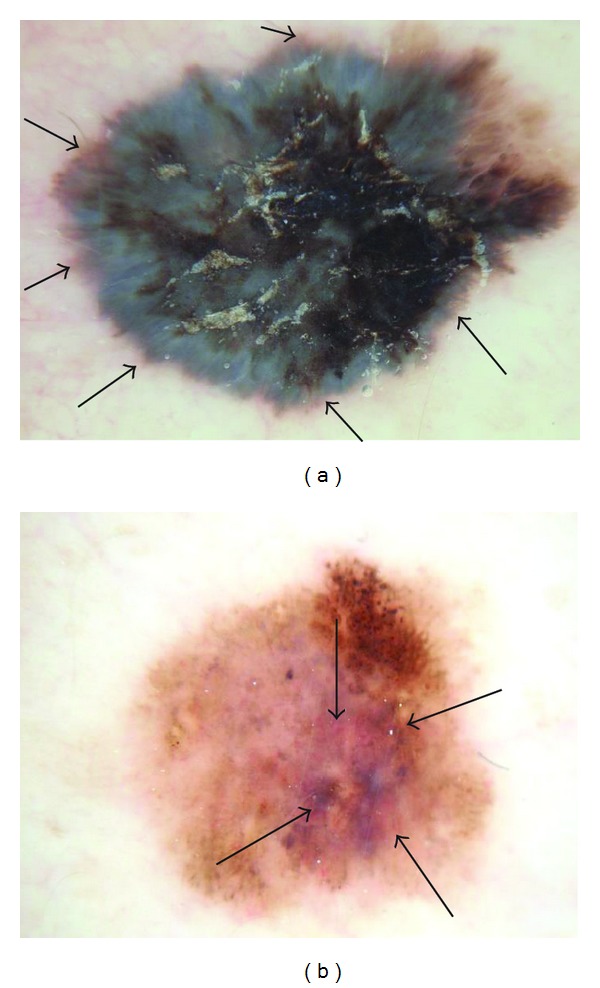
(a) Invasive melanoma, 10 mm wide, chest, 50-year-old male, 0.80 mm thick, peripheral-only pink, with circumferential pink rim sign (most prominent at arrows). (b) Clark compound nevus with moderate atypia, 5.5 mm wide, on shoulder, 25-year-old female. Note architectural disorder, irregularly distributed globules, atypical pigment net, and pink and blue color. Pink is most prominent at center (arrows), negative pink rim sign.

**Figure 5:**
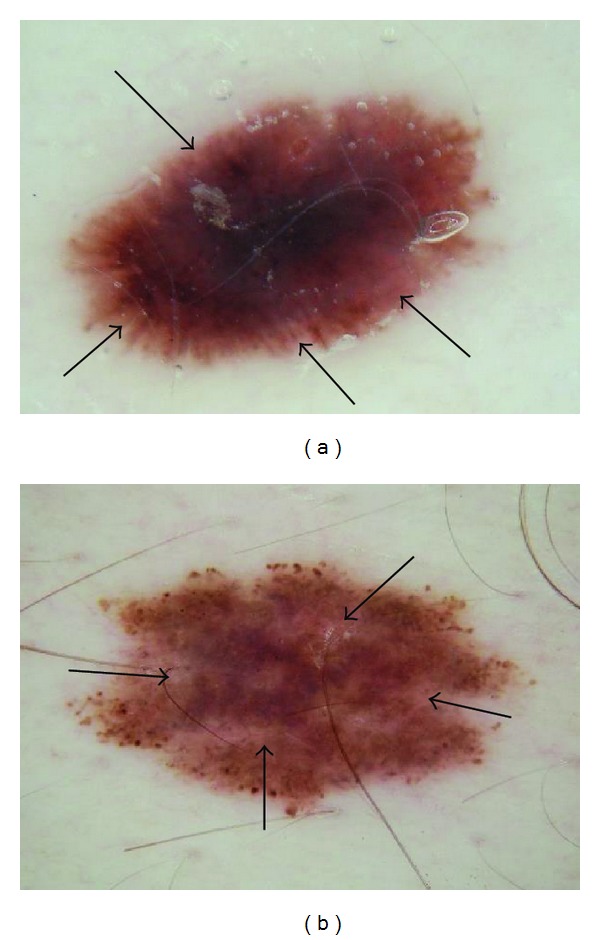
(a) Invasive melanoma, 5 mm wide, lateral abdomen, 32-year-old male, 0.44 mm thick, peripheral pink, with pink rim sign (arrows). (b) Enlarging melanocytic lesion with congenital features, 8 mm wide, abdomen, 21-year-old male. Pink is more prominent centrally than peripherally, negative pink rim sign.

**Figure 6:**
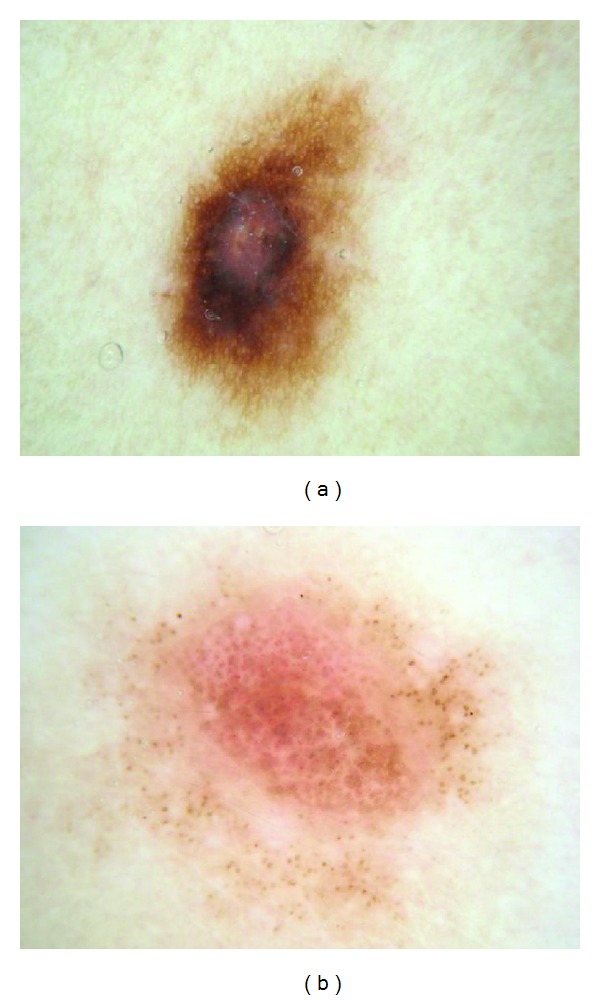
(a) Benign nevus. Central pink only. (b) Congenital nevus, no biopsy, central-only pink. In this nevus, pink extends nearly to the periphery but diminished at the far rim. Neither lesion was biopsied.

**Figure 7:**
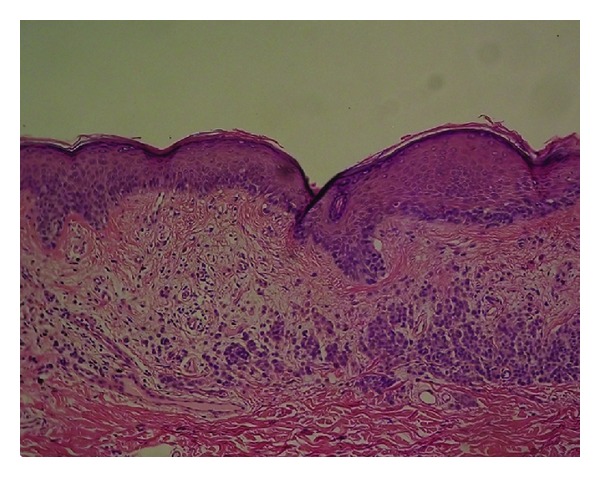
Histopathology for Figure 3(a), melanoma 0.55 mm depth.

**Figure 8:**
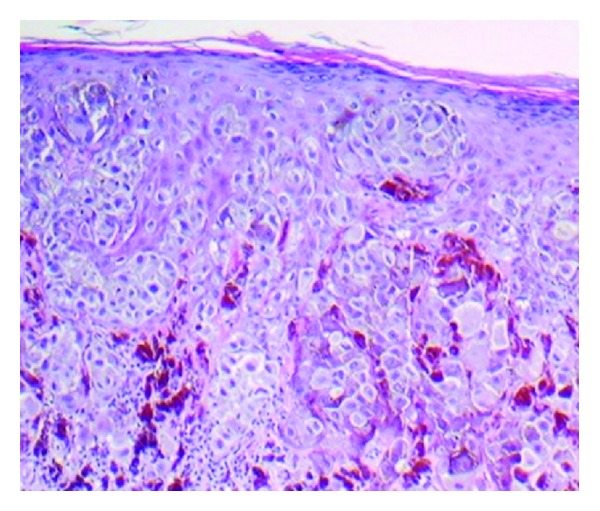
Histopathology for Figure 4(a), invasive melanoma 0.80 mm depth.

**Figure 9:**
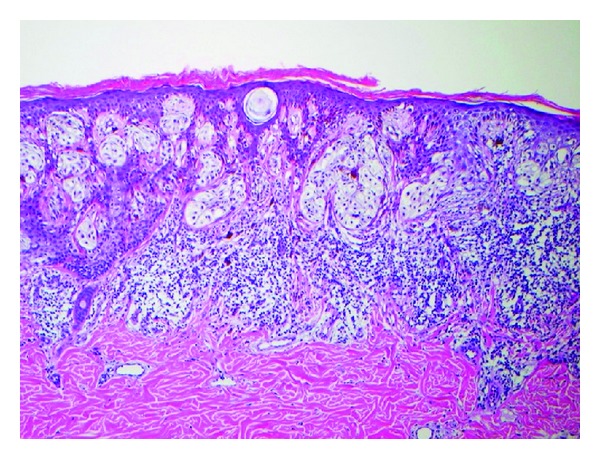
Histopathology for Figure 5(a), invasive melanoma 0.44 mm thick.

For peripheral pink, the pink color was present at the lesion rim in a large enough area to be easily detected (area diameter > 1 mm on 10 mm wide dermoscopic images) but need not be present circumferentially. Similarly, central pink was considered present if pink was present somewhere inside the lesion rim, occupying the same minimal area. Any discrepancies between the two observers were resolved by consensus on presence or absence of pink area. These findings were reviewed by a dermatologist (WVS). Figures 1–6 show dermoscopic views of malignant and benign lesions that had central pink, peripheral pink, or pink throughout the lesion. Histopathological imaging for Figures 3(a), 4(a), and 5(a) are seen in Figures 7, 8, and 9, respectively.

Sensitivity, specificity, and odds ratios were computed for the peripheral pink feature. The *P* values were computed from the chi-square distribution using the Wald chi-square value (Pearson uncorrected). Standard sensitivity and specificity were calculated as sensitivity: (true positive/(true positive + false negative)) and specificity: (true negative/(false positive + true negative)). Odds ratios were calculated as the odds of melanoma with peripheral pink present divided by the odds of melanoma without the feature: ((true positive × true negative)/(false negative × false positive)).

## 3. Results 

Of the entire pigmented lesion set, 1154/1290 (89%) had some pink in the lesion. Of the 296 malignant melanomas overall, 93% had pink color present in the lesion. The majority 899/1290 (70%) of lesions had pink color present somewhere in the lesion periphery. The invasive melanomas had a slightly higher percentage of peripheral-only pink (21/126 = 16.7%) compared to the *in situ* melanomas (22/170 = 12.9%). Of the 994 benign nevi, 64 (6.4%) had peripheral-only pink. The odds ratio for the presence of peripheral-only pink for all malignant melanomas is 2.51 (95% CI: 1.7–3.8, *P* < 0.0001) while the odds ratio for central-only pink is 0.462 (95% CI: 0.32–0.67, *P* = 0.204).

Pink color was present confined to the center of the lesion in 38/296 (12.8%) of melanomas and in 217/994 (21.8%) of benign lesions. Percentages of lesion types are shown in Table 1. A rim of pink at the outside of a pigmented lesion, in some cases even resembling the sun visible around the darker lunar object in a lunar eclipse or a rim of sun at sunset, can be termed the “pink rim sign.” The pink rim sign is present in the melanoma *in situ *in Figure 1(a) and the two invasive melanomas in Figures 4(a) and 5(a).

## 4. Discussion

The differentiation in the clinic of early malignant melanoma from benign pigmented lesions, even with dermoscopy, remains a difficult challenge. At the earliest stages, especially in melanomas lacking significant pigmentation, few dermoscopic clues may be present to aid in this differentiation. Irregular pigment network, negative network, atypical vascular patterns, and blue-gray granular peppering are helpful features if they are present [3, 7–11]. Yet these structures have specific patterns which can be challenging for those still learning dermoscopy. Pink color observed without magnification is a clinical clue for presence of malignancy, including primarily nonmelanocytic lesions, such as basal cell and squamous cell carcinoma [2]. Data presented here supports the hypothesis that any pink coloration observed dermoscopically confined to the periphery of a lesion increases the likelihood of melanoma (odds ratio 2.5), whereas pink observed only in the central, nonperipheral portion of the lesion decreases the likelihood of melanoma (odds ratio 0.46). We must indicate that any pink coloration includes a generous definition of pink. For example, pink-orange, light pink, and dark pink were all counted as pink. Menzies et al. noted that milky-red pink areas conferred an odds ratio of 2.5 for the diagnosis of melanoma among a group of amelanotic lesions and an odds ratio of 2.3 for the finding of more than one shade of pink [5]. Thus, our findings extend the findings of the Menzies group to most nevocellular lesions, with a similar odds ratio for peripheral pink. As our study demonstrates with dermoscopically naive observers, training in dermoscopy is not required for the detection of pink. In cases where other structures are absent, such as for hypomelanotic melanoma (Figure 3(a)) or symmetric lesions with few other features of melanoma (Figure 1(a)), the finding of pink color in the periphery can help lead to earlier excision.

The type of dermoscopy can affect results. Dermoscopic features related to vascularity, including pink color, can be altered by excess pressure caused by direct skin contact with the dermatoscope. However, our image set shows pink coloration preserved in the majority of contact dermoscopy images, demonstrating that contact dermoscopy can be a reliable detection method for pink coloration. In addition, Benvenuto-Andrade et al., in a study comparing dermoscopy techniques, showed greater preservation of pink veil by polarized dermoscopy [12]. However, our image set shows that with proper minimal-pressure technique using a gel interface, pink coloration is preserved in the majority of these contact dermoscopy images. Images in the Benvenuto-Andrade study show that pink coloration becomes redder in some non contact polarized dermoscopy images and appears in some benign images, creating a false-positive pink rim sign, such as what was seen in a dysplastic nevus and a blue nevus in that study [12]. We conclude that contact non polarized dermoscopy using proper technique, with gel and minimal pressure, gives the best color balance to allow reliable detection of the pink rim sign.

We may apply this clinical observation to analytic techniques to increase the accuracy of both computer-based diagnostic systems and human detection of melanoma. In computer-based systems, it is anticipated that combined color and location analysis can provide diagnostic assistance to practitioners. The pink rim sign might serve as an adjunct feature for such systems by applying the ratio of pink in the periphery to pink in the center of the lesion. For practitioners, in cases where it is difficult to determine whether structural features of melanoma are present, the finding of a pink rim sign can increase the index of suspicion for melanoma and may lead to earlier excision. Additionally, the presence of pink may be determined on clinical examination, without dermoscopy. Therefore pink serves as not only a dermoscopy warning sign but also an aid to localization of significant lesions.

At least two specific pink structures in dermoscopy have already been described. These include milky-red areas or globules [4] and pink veil [12]. Milky-red areas are vascular structures seen in hypomelanotic or amelanotic melanoma. Pink veil may be seen in benign lesions, including dermatofibroma, as well as basal cell carcinoma and melanoma [12]. Our findings for pink reflect those of Pizzichetta et al. for milky-red areas, who found for a group of benign and malignant amelanotic and hypomelanotic lesions that milky-red areas are present more frequently in melanomas (93% in thick melanomas; 31% in thin melanomas) versus 17% in benign lesions [4].

Our study avoids anyterminology specific to dermoscopy in defining “pink.” The three observers were not given any specific structural definition that they needed to learn but rather were instructed to just decide whether pink color was present at the lesion rim or center. Our definition of pink is more inclusive than “milky-red areas,” and more lesions will be described as pink. In our experience, nevi in people with fair skin (Fitzpatrick 1 and 2 skin types) generally contain pink color. The frequency of pink color observed in lesions will vary among populations. Thus the high frequency of pink color observed in our series cannot be generalized to all populations. We found that, in our thicker melanomas, for example, the 0.8 mm melanoma in Figure 4, pink is confined to the rim of the lesion because of more advanced central pigmentation, whereas in earlier melanomas pink was found throughout the lesion, as in the melanoma *in situ* in Figure 1 and the 0.55 mm melanomas in Figures 2 and 3. There is experimental support for the hypothesis that pink color in the periphery can indicate malignancy. Terushkin et al. showed that the blood volume visible by transillumination techniques exceeds the area of pigmented lesions seen on dermoscopy [13]. They also showed a correlation between the degree of atypia and the blood volume. On the spectrum from mild to moderate to severe atypia to malignant melanoma, blood volume was found to have been increased.

The finding of increased blood volume could help explain the reason that pink in the periphery is correlated with malignancy in pigmented lesions. We also found an increase in the presence of peripheral pink with more advanced diagnosis. Peripheral pink was present in 6.6% of nevocellular nevi, 12.9% of *in situ* melanomas, and 16.7% of invasive melanomas. This correlation did not extend to degree of atypia in benign lesions, however, as more nevi with mild atypia (5.64% had peripheral pink than those with moderate atypia (2.86%)). Otherwise, the progressive increase in blood volume with increasing atypia observed in the Terushkin study correlates overall with our observed increase in peripheral pink percentages as well as the progression of milky-red areas in the Pizzichetta study (93% in thick melanomas; 31% in thin melanomas) versus 17% in benign lesions [4]. The findings of the Terushkin transillumination study provide a physiologic reason why pink in the periphery may have more discriminatory power for any skin cancer than pink in other areas.

Although pink is correlated with erythema, vascularity, and blood volume, there is no 1 : 1 correlation. Pink is a more inclusive and less specific criterion. The advantage to pink as a sign for melanoma is that it can be detected by naive observers.

Our study is limited by the nonconsecutive acquisition of a convenience sample of lesions in the four clinics above, with clinical and dermoscopic digital photographs obtained between 2007 and 2011. These lesions comprise the majority of melanomas found in these clinics during this interval. For this study, we eliminated two of the most difficult diagnostic categories of lesions—collision tumors and melanocytic lesions with high-grade dysplasia. The first category, collision tumors, those difficult lesions that can show features of both benign and malignant lesions, must be analyzed piecewise, requiring advanced dermoscopy skills. For the second category, high-grade dysplastic lesions, the analysis can be complicated by disagreement among dermatopathologists. Another limitation present in our study is that we employed only student observers, neither of whom was formally trained in dermoscopy. These nonphysician observers may nonetheless model the skills of physicians beginning training in dermoscopy, and, as dermoscopy is more widely employed by nonspecialists, the pink rim sign could be used by these physicians.

Another limitation is the possibility of unconscious introduction of bias, using a different technique such as applying less central pressure when acquiring melanoma images, resulting in more apparent erythema in the periphery. Nevi have less erythema and when capturing the image the photographer would not likely have paid attention to the amount of pressure being applied resulting in the vasculature being blanched. As this is a pilot study, future studies are needed to confirm this observation. Future studies could be done with noncontact dermoscopy, and lesions could be evaluated by those with experience in dermoscopy to further explore these findings.

## 5. Conclusion

A pink rim in a melanocytic lesion is a clue to the diagnosis of melanoma. An odds ratio of 2.5 was calculated for any shade of pink confined to the periphery of dermoscopic images of melanocytic lesions. We did not restrict the shade of pink. Pink-orange and dark pink were considered pink. These pink areas can be detected by people without formal dermoscopic training. This pink color likely corresponds to increased blood volume. When pink color is observed in the periphery of a dermoscopic image of a melanocytic lesion, this feature suggests consideration of melanoma within the differential diagnosis.

## Figures and Tables

**Table 1:** Number of lesion types by location of pink coloration.

Lesion type	Peripheral-only pink (lesion type total)	Central-only pink	Pink throughout entire lesion
Invasive melanoma*	21 (126); 16.7%**	15 (126); 11.9%	80 (126); 63.5%
Melanoma *in situ *	22 (170); 12.9%	23 (170); 13.5%	115 (170); 67.7%
Nevus without atypia	18 (271); 6.6%	54 (271); 19.9%	181 (271); 19.9%
Dysplastic nevus, mild atypia	26 (461); 5.6%	103 (461); 22.3%	268 (461); 58.1%
Dysplastic nevus, moderate atypia	2 (70); 2.86%	13 (70); 18.6%	48 (70); 68.6%
Congenital nevus	18 (192); 9.4%	47 (192); 24.5%	101 (192); 52.6%

*Median Breslow depth: 0.41 mm. **Differences in lesion totals represent lesions with no pink.
